# Divergent structure–function coordination shapes leaf thermal tolerance in Mediterranean tree species

**DOI:** 10.1111/plb.70289

**Published:** 2026-09-14

**Authors:** L. Friulla, L. M. Iozia, M. F. Crescente, L. Varone

**Affiliations:** ^1^ Department of Environmental Biology Sapienza University of Rome Rome Italy

**Keywords:** *Acer campestre*, *Fraxinus ornus*, PSII thermal stability, *Quercus ilex*, T50, urbanity, Tcrit

## Abstract

- Increasing temperatures impair photosynthetic apparatus by damaging photosystem II (PSII) efficiency. We evaluated the relevance of two thermal indices (Tcrit and T50) as indicators of the PSII resistance to thermal stress of *Quercus ilex*, *Acer campestre* and *Fraxinus ornus* in the urban area of Rome.
- Tcrit (critical temperature) and T50, that is the temperature at which the maximum PSII efficiency (Fv/Fm) decreased by 15% and 50%, were estimated by modelling Fv/Fm response curves of leaves exposed to the temperature range of 25–60 °C. To evaluate the functional relevance, Tcrit and T50 data were integrated with leaf functional traits using a multivariate analysis approach.
- *Q. ilex* exhibited a lower Tcrit than *A. campestre* and *F. ornus*. T50 was similar between species. In *Q. ilex*, early Fv/Fm decline was consistent with an early photoprotective downregulation rather than reduced heat resistance, with PSII activity persisting even at extreme temperatures. *A. campestre* and *F. ornus* maintained high Fv/Fm up to Tcrit rapidly collapsing beyond this threshold. The contribution of physiological and morphological traits to thermal resistance was both metric‐dependent and species‐specific. Morphological and physiological traits were primarily tied to Tcrit and T50, respectively. In *Q. ilex*, morphological traits explained a greater variance in both thermal indices, while physiological traits dominated in *A. campestre*, and a shared physiological–morphological contribution prevailed in *F. ornus.*
- Thermal resistance arose from the coordinated interplay between structure and function, reflecting distinct species‐specific ecological strategies.

## Graphical abstract

Tcrit (critical temperature) may reflect early, adaptive photoprotective downregulation of PSII in *Quercus ilex*, whereas in *Acer campestre* and *Fraxinus ornus*, it may more closely correspond to the onset of thermal PSII vulnerability.

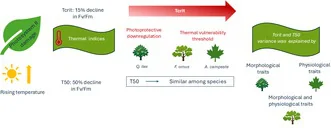

## INTRODUCTION

Heat or thermal stress is a major driver of impairment to the photosynthetic apparatus, especially at the expense of photosystem II (PSII) (Berry & Bjorkman 1980). Thermal stress is defined as a rise in temperature that exceeds a critical physiological threshold of tolerance over a sustained period, resulting in serious and sometimes irreversible damage to PSII (Percival 2023). Given the high sensitivity of the PSII to heat, thermal stress is usually quantified by chlorophyll fluorescence–based indices (Perez & Feeley 2020; Martínez‐Villa *et al*. 2024; Zhang *et al*. 2024), which measure the percentage of damage inflicted on the maximum PSII efficiency (Fv/Fm).

Plant thermal tolerance is a complex phenomenon controlled by multiple physiological processes, including the synthesis of zeaxanthin and other antioxidants, adjustments in membrane fatty acid composition, the induction of heat shock proteins and hormonal regulation (Kullberg & Feeley 2024).

The predictive capacity of the chlorophyll fluorescence–based indices in assessing leaf vulnerability may be enhanced by integrating these indices with functional leaf traits that directly reflect the physiological mechanisms underlying thermal tolerance. These mechanisms include the accumulation and regulation of photosynthetic pigments such as chlorophyll and carotenoids, as well as secondary metabolites. Leaf spectral reflectance indices, including the normalized difference vegetation index (NDVI), carotenoid reflectance index (CRI), anthocyanin reflectance index (ARI) and photochemical reflectance index (PRI), provide effective tools for detecting early plant stress responses (Katsoulas *et al*. 2016). These indices capture changes in chlorophyll content, antioxidant pools and xanthophyll‐cycle activity, thereby establishing a mechanistic link with the biochemical and photoprotective systems regulating the PSII resistance to thermal stress.

However, morphological and anatomical traits such as leaf mass per area (LMA), leaf area (LA), leaf thickness (LT) and leaf tissue density (LTD) also play a pivotal role in shaping species‐specific responses of PSII to thermal stress (Valliere *et al*. 2023; Mitchell *et al*. 2025) because they influence the leaf capacity to regulate temperature, reduce water loss and dissipate excess heat. For instance, smaller leaf areas limit direct exposure to sunlight, reducing heat absorption, while greater tissue density and increased thickness enhance insulation, preventing rapid temperature fluctuations within the leaf (Valliere *et al*. 2023; González *et al*. 2025). These traits collectively improve plant capacity to maintain cellular function and water balance under high temperature conditions, increasing overall survival and performance in heat‐stressed environments. Nevertheless, empirical evidence indicates that these relationships are context‐dependent, varying across leaf habits, habitats and climatic gradients, which highlights the limitations of relying on single‐trait predictors (Zhang *et al*. 2024).

The study of plant resilience to thermal stress assumes a particular relevance in urban areas and even more so in cities characterized by a Mediterranean climate. While the effects of climate change are serving to intensify the well‐known urban heat island phenomenon (Oke 1982; Percival 2023; Martínez‐Villa *et al*. 2024), they also exacerbate the intrinsic ecological vulnerability of the Mediterranean Basin, which is recognized as a climate‐vulnerable region (Valliere *et al*. 2023). The Mediterranean plant species have evolved specific features to face the arid summer conditions resulting from high temperatures, intense solar radiation and limited soil water availability (Larcher 2000). These adaptations include the development of sclerophyllous evergreen leaves, characterized by high LMA and LT, that limit leaf transpiration to minimize water loss (Gratani & Varone 2004). Nonetheless, these structural adaptations also lead to storing more heat, often resulting in leaf temperatures that exceed ambient air temperatures (Larcher 2000). Moreover, Mediterranean deciduous tree species exhibit more thermophilic characteristics than their non‐Mediterranean counterparts (Peguero‐Pina *et al*. 2016), indicating that thermal adaptation is not limited to evergreen species.

Expanding our understanding of the thermal PSII stability of plants living in Mediterranean cities is essential. This knowledge is crucial not only for predicting their capacity to withstand increasingly severe temperature conditions but also for more effective planning of resilient green spaces. Such planning can help sustain biodiversity and enhance the urban microclimate under worsening thermal stress.

This study aimed to evaluate the thermal tolerance of three Mediterranean species commonly found in the city of Rome. Our focus was specifically on the thermal resistance of the photosynthetic apparatus, which was assessed through PSII thermal stability indices, rather than on heat‐avoidance mechanisms such as transpiration‐driven evaporative cooling.

A pertinent index of the PSII thermal stability is Tcrit. Several operational definitions of Tcrit have been proposed, including breakpoints in basal fluorescence Fo to temperature relationship (Dongsansuk *et al*. 2013), the intersection between the slow and fast rise phases of Fo with rising temperature (Knight & Ackerly 2002), and the inflection points or slope changes in chlorophyll fluorescence curves (Geange *et al*. 2020). Other protocols define Tcrit as the temperature at which Fv/Fm declines by 15% (Perez & Feeley 2020; Zhang *et al*. 2024). Our study is based on this last definition, which has some advantages compared with other methods. First, it reflects an early indication of reduced PSII functionality still reversible (Didion‐Gency *et al*. 2025). Second, as Tcrit is estimated from a continuous sigmoidal fit to the Fv/Fm ‐ T response curve, it allows for avoiding subjective line‐fitting and largely avoids the sample‐exclusion problem of F_O_‐based protocols (Andrew *et al*. 2023). Finally, this definition of Tcrit maximizes cross‐study comparability, since Fv/Fm is the most widely validated proxy for PSII efficiency under both field and laboratory conditions (Neri *et al*. 2024).

In addition to Tcrit, we also used T50, that is the temperature corresponding to a 50% reduction in Fv/Fm, as a PSII thermal stability indicator. This threshold is indicative of potentially irreversible PSII damage (Zhang *et al*. 2024).

Moreover, we applied an integrative approach that combines functional reflectance‐based indices with morphological and anatomical traits to provide a comprehensive assessment of the functional relevance of Tcrit and T50.

Our specific objective was to identify the strategies that shape species‐specific resilience to thermal stress. To hit the goal, we selected two deciduous species (*Acer campestre, Fraxinus ornus*) and one evergreen species (*Quercus ilex*). Evergreen sclerophylls invest in long‐lived, structurally robust leaves that prioritize resource conservation, while deciduous species develop thinner, short‐lived leaves that emphasize rapid physiological responses (Richardson *et al*. 2014).

By comparing these two strategies, we wanted to better understand the diversity of Mediterranean species and take this opportunity to also explore the possibility that structural investment or physiological flexibility could provide greater resilience under extreme temperatures.

We hypothesize that the relative contribution of morphological and physiological traits to PSII thermal tolerance differs between *Q. ilex* and *A. campestre*, and *F. ornus* due to their different leaf habitus. Specifically, we predicted that the evergreen *Q. ilex* would show a stronger association between thermal tolerance and structural leaf traits, whereas the deciduous *A. campestre* and *F. ornus* would rely more strongly on physiological traits. Consequently, we expected variation in Tcrit and T50 to reflect different structure–function coordination strategies among species.

## MATERIAL AND METHODS

### Study area and plant species

The study was carried out in the city of Rome from 1 July to 31 July 2024. Rome is characterized by a Mediterranean type of climate. Data collected over a long period (2013–2023; data from the Latium Regional Agency for Development and Agricultural Innovation; Meteorological Station of Rome, Lanciani Street) revealed an average total yearly rainfall of 855.60 mm, most of it distributed in autumn and winter. The average maximum air temperature of the hottest months (July and August) was 32.5 ± 1.2 °C, and the average minimum air temperature of the coldest month (January) was 4.6 ± 1.3 °C. The mean yearly air temperature was 17.3 ± 6.7 °C.

However, in recent years, Rome's climate seems to be becoming increasingly hotter, with average temperatures rising, especially in the summer months (ISTAT 2024; www.istat.it/wp‐content/uploads/2024/06/Tavole_Dati‐Meteoclimatici_Capoluoghi‐Provincia_Anno‐2022‐e‐serie‐2006‐2022_DCAT.xlsx). This trend was observed during the study period, since July 2024 was characterized by an average maximum air temperature of 34.6 ± 2.3 °C, an average minimum air temperature of 23.5 ± 1.7 °C and by an average temperature of 29.1 ± 7.8 °C, and a total average rainfall of 0.7 mm (data from the Latium Regional Agency for Development and Agricultural Innovation; Meteorological Station of Rome, Lanciani Street, https://siarl.arsial.it/bi/superset/dashboard/18/).

The species selected for this study (*A. campestre, F. ornus* and *Q. ilex*) were widely distributed in the city of Rome (Attorre *et al*. 1997; Gratani *et al*. 2016).

*A. campestre*, a deciduous mesophilic species, has a broad geographical and adaptive distribution, ranging from continental Eastern Europe to the Maghreb (Schumann *et al*. 2019). It is a subdominant species involved in the formation of mixed temperate deciduous forests (Zecchin *et al*. 2016), and it is often used as an ornamental tree in urban parks and gardens, as well as in the construction of hedges in urban green spaces (Wahlsteen *et al*. 2023). *F. ornus* is a Mediterranean deciduous species commonly found in temperate hills and mountain forests in the Mediterranean region (Caudullo & de Rigo 2016). It is highly adaptable, thriving in both xeric and alpine environments, as well as in areas with higher water availability (Kalapos & Csontos 2003; Petruzellis *et al*. 2018). *Q. ilex* is an evergreen sclerophyll, widely distributed across the Mediterranean basin. Although it is typically xerophilic, it can also be found in mesophilic environments (Archibold 1995).

We intentionally included two deciduous species and just one evergreen to capture the higher intra‐group variability in the deciduous strategy. Deciduous tree species, being structurally less constrained than evergreen species, display a higher resource acquisition plasticity (Boehnke & Bruelheide 2013). On the contrary, *Q. ilex* may be considered a reference species in the Mediterranean region and a well‐studied model of the sclerophyllous strategy, so its inclusion alone was sufficient to represent the evergreen functional type. Moreover, it is considered a target species in Mediterranean urban areas (Fusaro *et al*. 2021).

For each species, 12 individuals (3–4 years old at the time of sampling) were selected. Whole‐plant structural traits including tree height (H, m), trunk diameter at 1.30 m (DBH, cm) and Leaf Area Index (LAI) were measured for each individual per species. H was measured by a laser telemeter (Forestry Pro II Nikon, Japan), DBH by a calliper and LAI by a LAI‐2000 Plant Canopy Analyser (LI‐COR, USA).

H, DBH and LAI equalled, respectively, 5.36 ± 0.34 m, 11.1 ± 0.3 cm and 2.33 ± 0.35 for *A. campestre*; in *F. ornus*, H, DBH and LAI were 3.89 ± 0.20 m, 6.3 ± 0.1 cm and 2.27 ± 0.37; and in *Q. ilex*, 5.53 ± 0.21 m, 14.3 ± 0.6 cm and 3.10 ± 0.40, respectively. The study intentionally focused on young trees because they had been planted as part of an urban afforestation project. Investigating trees at this developmental stage provides valuable insights into their establishment and early performance under urban environmental conditions.

The sampled leaves were fully developed and south facing; sampling was conducted in July between 12:00 and 14:00, under a mean maximum temperature of 34.1 ± 2.9 °C.

### Thermal tolerance

Thermal tolerance was evaluated in terms of Tcrit and T50 according to Zhang *et al*. (2024) on 36 leaves with a south‐east orientation collected from each individual per species by a tree pruner (3 leaves per individual). Once the leaves were collected, they were carefully placed into sealed plastic bags, each labelled with the corresponding tree's identification. To ensure the leaves remained fresh, the petioles were gently inserted into paper towels that had been moistened with cold water. These bags were then stored in a cooler to preserve the leaf condition and transported to the laboratory in 15–20 min.

In the laboratory, leaves were subjected to a two‐hour dark‐adaptation period using leaf clips designed for chlorophyll fluorescence measurements, which kept the measured leaf area in darkness until the saturating light pulse was applied. Moreover, during this period, leaves were maintained with their petioles immersed in water inside a beaker and kept at a constant temperature (25 °C) in a temperature‐controlled chamber. In addition, the leaves were covered with aluminium foil to further minimize water loss and maintain leaf hydration. After the two hours of dark adaptation, the maximum PSII efficiency (Fv/Fm) was measured by a Fluorpen FP 110 fluorometer (Photon Systems Instruments, Czech Republic). This initial Fv/Fm measurement enabled the assessment of the health condition of the leaves before the thermal treatment application.

After the initial measurement, the leaves were stored with their stems immersed in water inside a beaker and placed in a temperature‐controlled chamber overnight as described above. The following morning, thermal treatments were initiated using a water bath (Argolab WB mod. 22 l, Italy). A total of eight different temperature treatments were set up (25, 30, 35, 40, 45, 50, 55 and 60 °C), according to Zhang *et al*. (2024). To exclude any time‐dependent decline in leaf function, Fv/Fm was measured every 15 min for 120 min before the thermal treatments (Fig. S1).

To avoid cumulative stress and repeated exposure effects, a discrete treatment design was employed. For each species, the total pool of leaves was randomly divided into eight independent groups, each assigned to a single temperature level. Each group was composed of 4–5 leaves and placed within a sealed plastic bag, ensuring that no water came into direct contact with them, and was submerged in the water bath. Moreover, air was removed from the bags according to Perez & Feeley (2020) to minimize air volume and promote efficient temperature equilibration between the water bath and the leaf tissue. Each thermal treatment lasted for 15 min. After each treatment, the leaves were stored in the refrigerator at 25 °C and kept in the dark for 24 h as described above. Subsequently to this period, Fv/Fm was measured again for each thermal treatment to quantify leaf damage due to the heat treatments (Feeley *et al*. 2020; Neuner & Buchner 2023; Zhang *et al*. 2024).

Tcrit and T50 were estimated for each species by modelling the Fv/Fm values *versus* temperature treatment. To model the Fv/Fm temperature response, three different logistic‐based probability distributions were compared: a type‐2 Weibull distribution, a third‐order sigmoidal logistic function and a second‐order sigmoidal logistic function, all computed in the R software environment (R Core Team). The Weibull function was obtained from the *fitplc* package (Zhang *et al*. 2024), while other curves were computed using the *nls* function from the *stats* package (Perez & Feeley 2020), and the *drm* function from the *drc* package (Valliere *et al*. 2023) using the self‐starting algorithms for nonlinear regression collected in the *aomisc* package. The adequacy of the model fits was evaluated by comparing the mean square error and overall goodness of fit. Based on this comparison, the Weibull function was thus selected as the most accurate and reliable model for estimating the thermal tolerance indices in this study.

### Leaf physiological and morphological traits

An additional sampling was carried out to measure leaf physiological and morphological traits. These traits were measured on 5 leaves per individual and species (60 leaves per species), which were comparable in size and crown position to those used in thermal tolerance measurements.

Leaf physiological traits were based on the reflectance indexes; they included: the Normalized Difference Vegetation Index (NDVI), the Photochemical Reflectance Index (PRI), the Anthocyanin Reflectance Index (ARI1), the Carotenoid Reflectance Index (CRI1), the Normalized Pigment Chlorophyll Index (NPCI) and the Water Index (WI).

These indices were measured in the field, by using two‐handled spectrophotometers (Polypen RP 410/UVIS and RP 410/NIR, Photon Systems Instruments, Czech Republic), respectively, with a spectral range of 380–790 nm (RP 410/UVIS) and of 640–1050 nm (RP 410/NIR). In addition, the amount of chlorophyll was measured as chlorophyll index (CI, SPAD units) using a chlorophyll meter (502 Plus, Konica Minolta, Japan).

The leaf morphological traits, such as leaf area (LA, cm^2^), leaf thickness (LT, μm) and leaf dry mass (DM, g), were measured on the same leaves used for physiological traits. LA was measured by using an image analysis system (Delta‐T Devices, Cambridge, UK). LT was measured via a digital micrometre (Kennedy, UK). DM was measured by using an analytical balance (JK‐180, CHIYO, Japan) on leaves dried in a thermostatic oven (M 710, GALLI, Italy) at 75 °C for 3 days. Furthermore, leaf mass per area (LMA, mg cm^−2^) was calculated as the DM/LA ratio and leaf tissue density (LTD, mg cm^−3^) as the LMA/LT ratio.

### Statistical analysis

Given the normality of data and accounting for heteroscedasticity, Welch's ANOVA was used to test for differences between groups, followed by post‐hoc Tukey tests to study interspecific variations in Tcrit and T50. However, physiological and morphological traits were not normally distributed so, to test for differences within that dataset, we used a set of pairwise Kruskal–Wallis tests. A principal component analysis (PCA) was performed based on the correlation matrix to summarize the multivariate pattern of variation in functional traits and thermal indices across species.

Since the study aim was to disentangle the relative contribution of physiological *versus* morphological traits in explaining variation in thermal tolerance indices, a variation partition analysis (VPA) was carried out. The VPA aimed to quantify the proportion of variance in Tcrit and T50 that was attributable to physiological and morphological traits, as well as their joint contribution. The significance of testable individual and shared partitions was evaluated through a Monte Carlo permutation test of the predictor effect. VPA was carried out both on the entire dataset and separately for each species to account for species‐specific strategies.

All analyses were conducted with the R Software (R Core Team, version 4.3.2).

## RESULTS

### Thermal tolerance

Fv/Fm generally declined with increasing temperature across all species (Fig. 1), although the onset and magnitude of this decline varied among them, highlighting species‐specific responses. *A. campestre* and *F. ornus* maintained high and relatively stable Fv/Fm values at temperature steps 25–45 °C, showing only small changes or occasional slight recoveries. At higher temperatures, however, their responses diverged: *A. campestre* exhibited a stronger decline than *F. ornus* between 45 and 50 °C, whereas above 50 °C, the reduction in Fv/Fm was more pronounced in *F. ornus*.

**Fig. 1:**
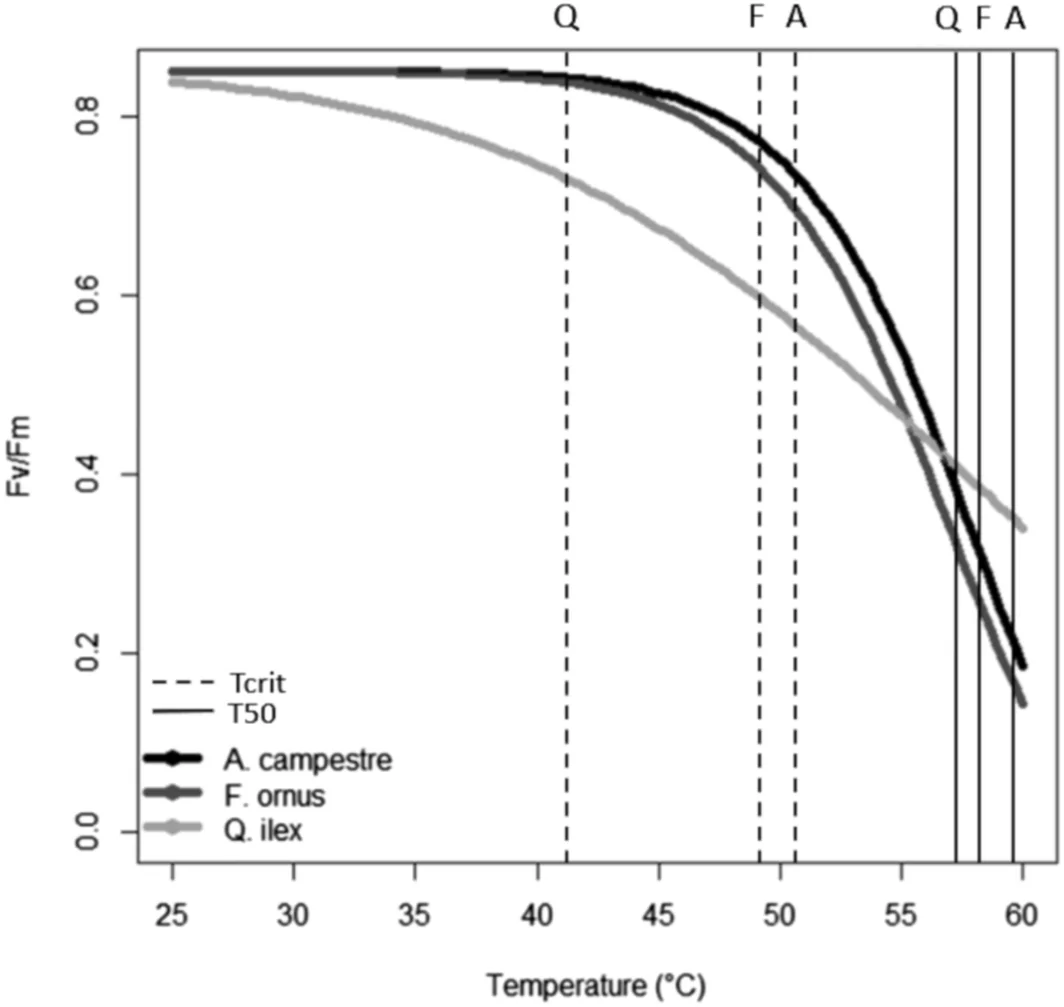
Fv/Fm response curve for the eight temperature treatments (25, 30, 35, 40, 45, 50, 55, and 60 °C) for *Q. ilex* (light‐grey curve), *A. campestre* (black curve) and *F. ornus* (dark‐grey curve). The dotted line and the solid lines represent the temperatures at which a 15% (Tcrit) and a 50% (T50) decrease in Fv/Fm occur, respectively. The letters Q, F and A correspond to *Q. ilex* (Q), *F. ornus* (F) and *A. campestre* (A). The Fv/Fm values *versus* temperature treatment were modelled by the Weibull logistic‐based function (n = 36 leaves for species).

By contrast, *Q. ilex* showed signs of thermal sensitivity at lower temperatures compared with the other species, with changes in Fv/Fm already evident around 30–35 °C, followed by a more gradual decline. Nevertheless, the magnitude of Fv/Fm reduction at the highest temperatures was lower than in *A. campestre* and *F. ornus*.

In line with the Fv/Fm responses, *Q. ilex* exhibited a significantly lower Tcrit (42.1 ± 3.6 °C) than *A. campestre* (50.9 ± 2.4 °C) and *F. ornus* (49.1 ± 4.1 °C). By contrast, no significant interspecific differences were found for T50 (*Q. ilex* 57.5 ± 4.6 °C, *A. campestre* 59.9 ± 1.1 °C, *F. ornus* 55.8 ± 1 °C), although a trend of variation among the three species in T50 values was observed.

### Leaf physiological and morphological traits

Physiological and morphological leaf traits were described using median values (Fig. 2).

**Fig. 2:**
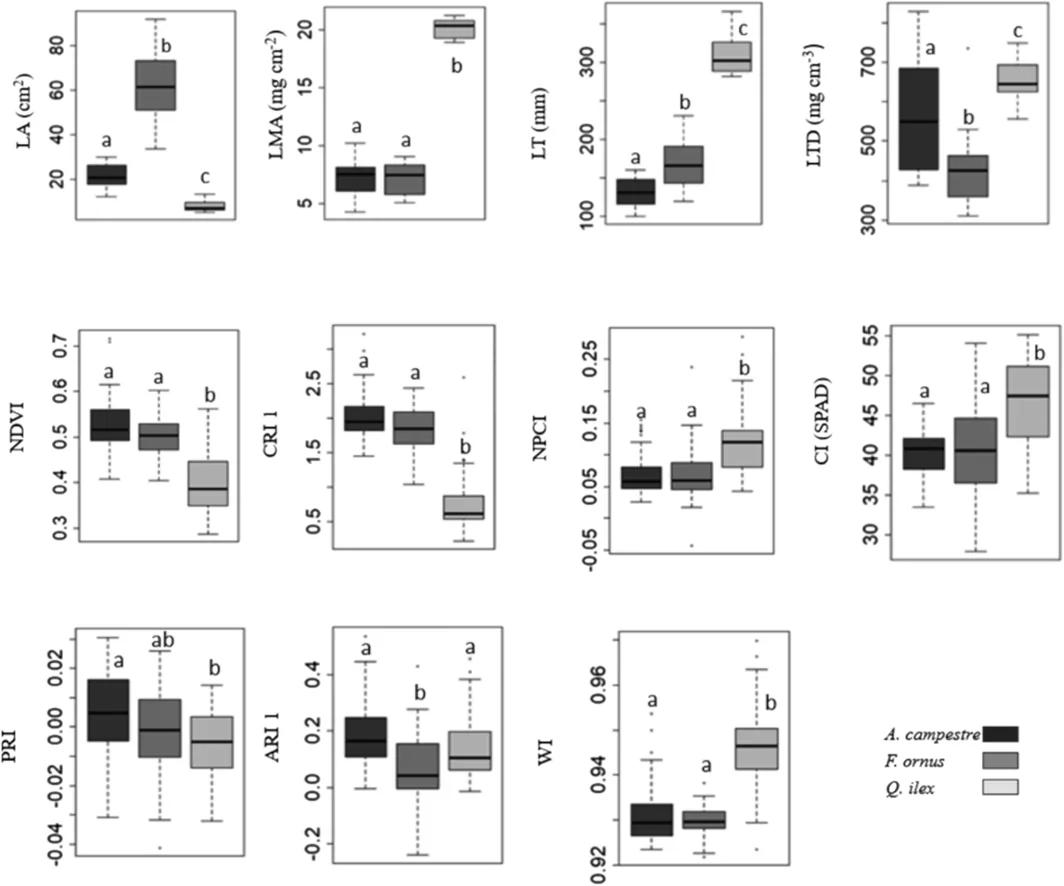
Median values and interquartile ranges (25th–75th percentiles) of the anthocyanin reflectance index (ARI1), carotenoid reflectance index (CRI1), chlorophyll index (CI), leaf area (LA), leaf mass per area (LMA), leaf thickness (LT) and leaf tissue density (LTD), normalized difference vegetation index (NDVI), normalized pigment chlorophyll index (NPCI), photochemical reflectance index (PRI) and water index (WI), measured in the studied species. Different letters indicate statistically significant differences among species (pairwise Kruskal–Wallis test; *P* ≤ 0.05, n = 60 leaves per species).

Physiological traits exhibited significant interspecific differences, primarily separating *A. campestre* and *F. ornus* from *Q. ilex*. Median values for most physiological traits did not differ significantly between *A. campestre* and *F. ornus*, whereas significant differences were observed between these species and *Q. ilex*.

NDVI values were higher in *A. campestre* and *F. ornus* (0.52 and 0.50, respectively) than in *Q. ilex* (0.39). A similar pattern was observed for CRI1, with higher and comparable values in *A. campestre* and *F. ornus* (1.95 and 1.85) and significantly lower values in *Q. ilex* (0.61).

The PRI value was lowest in *Q. ilex* (− 0.0050) and highest in *A. campestre* (0.0048), while *F. ornus* showed intermediate values that did not differ significantly from either species. By contrast, ARI1 exhibited an opposite trend, with no significant differences between *A. campestre* and *Q. ilex* (0.165 and 0.105, respectively), whereas *F. ornus* showed significantly lower values (0.042).

WI and CI were significantly higher in *Q. ilex* (0.95 and 47.5 SPAD units, respectively) than in *A. campestre* and *F. ornus*. Despite its higher chlorophyll content, *Q. ilex* also showed a greater proportion of total leaf pigments relative to chlorophyll, as indicated by the highest median NPCI value (0.120), compared with *A. campestre* (0.059) and *F. ornus* (0.058).

Unlike physiological traits, morphological traits displayed greater interspecific variation that was not strictly related to leaf habit. Except for LMA, which did not differ between *A. campestre* and *F. ornus* (7.5 mg cm^−2^), all morphological traits differed significantly among *A. campestre*, *F. ornus* and *Q. ilex*, with this latter exhibiting the highest values of LMA (20.4 mg cm^−2^), LT (303 mm) and LTD (645 mg cm^−3^), while showing the lowest leaf area (7.3 cm^2^).

### Multivariate analysis

To assess how physiological and morphological traits jointly shape thermal tolerance, we adopted a multivariate approach that incorporated both principal component analysis (PCA) and variation partitioning analysis (VPA). Specific information on the VPA is provided in Tables S1–S4.

The PCA extracted three main components, PC1, PC2 and PC3, explaining 72% of the total variance (Table 1).

**Table 1:** Results of the principal component analysis (PCA) via correlation matrix carried out on the considered physiological and morphological leaf traits across species.

trait	PC1 (47.0%)	PC2 (13.6%)	PC3 (10.6%)
NDVI	**−0.81**	0.0059	**0.40**
PRI	**−0.45**	**0.65**	**0.42**
ARI1	**0.18**	**−0.82**	**0.24**
CRI1	**−0.88**	**−0.23**	**0.19**
WI	**0.84**	0.03	−0.06
NPCI	**0.66**	**−0.39**	−0.14
CI	**0.52**	**0.42**	**0.20**
LA	**−0.67**	**0.18**	**−0.46**
LMA	**0.92**	**0.22**	0.13
LT	**0.85**	**0.38**	−0.08
LTD	**0.55**	**−0.21**	**0.53**
T50	**0.27**	0.03	**0.57**
Tcrit	**−0.78**	0.06	**0.16**

The percentage of variance explained by each principal component (PC) and the correlation between the selected variables and the PCs are shown. Pearson's pairwise correlation coefficients presented in bold were significant at *P* ≤ 0.05.

ARI1 = anthocyanin reflectance index; CI = chlorophyll index; CRI1 = carotenoid reflectance index; LA = leaf area; LMA = leaf mass per area; LT = leaf thickness; LTD = leaf tissue density; NDVI = normalized difference vegetation index; NPCI = normalized pigment chlorophyll index; PRI = photochemical reflectance index; T50 = temperature at which Fv/Fm decreases by 50%; Tcrit = temperature at which Fv/Fm decreases by 15%; WI = water index.

Most of the variance (47.0%) was explained by PC1; however, despite a lower contribution of PC2 (13.6%) and PC3 (10.6%), these components provided some useful insights to understand how the functional traits covariate with Tcrit and T50.

PC1 was positively correlated with LMA, LT, WI, NPCI, SPAD, LTD and negatively correlated with NDVI, CRI1, LA and Tcrit (Table 1).

Along the PC1, the biplot (Fig. 3A,B) showed a clear separation among the studied species, with *A. campestre* and *F. ornus* having larger and thinner leaves than *Q. ilex*.

**Fig. 3:**
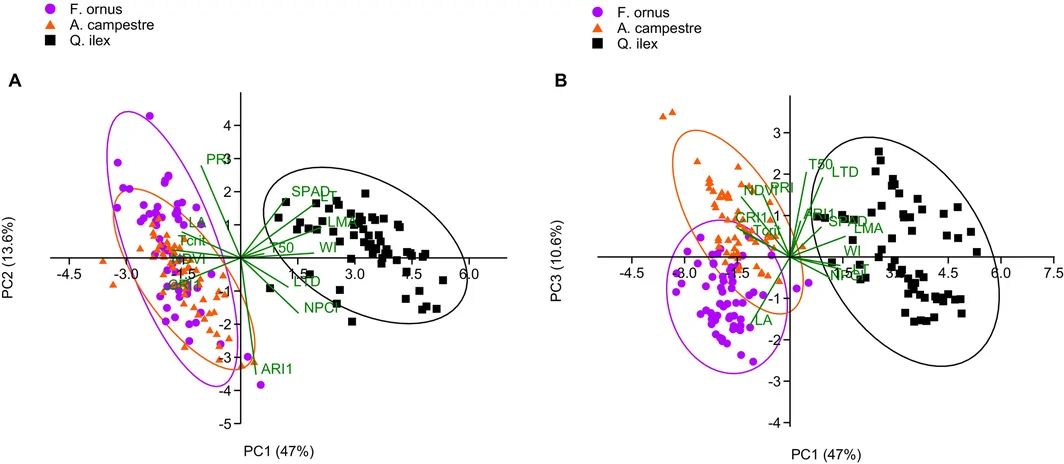
Principal component analysis (PCA) biplots. (A) PC1 *versus* PC2; (B) PC1 *versus* PC3. The percentage of total variance explained by each PC is shown.

PC2 (Fig. 3A) showed a strong positive correlation with PRI and a negative correlation with ARI1, as well as a weaker but still significant correlation with SPAD and NPCI. Therefore, PC2 reflected leaf photochemical activity and pigment composition.

PC3 (Fig. 3B) was strongly and positively correlated with LTD and T50, suggesting that LTD is a key factor in slowing irreversible damage to PSII. Along PC3, *A. campestre* and *Q. ilex* exhibited similar behaviour, achieving higher scores than *F. ornus*, the species in the most disadvantaged position (Fig. 3B).

To first assess the overall pattern across species, a VPA was performed on the entire dataset; species‐specific VPAs were subsequently conducted to evaluate how the relative contribution of physiological and morphological traits differed among species. When all species were considered together (Fig. 4), a greater proportion of variance was explained for Tcrit than for T50 (overall residual variance for Tcrit: 0.35; for T50: 0.82), irrespective of whether variance was attributed to physiological traits (PT), morphological traits (MT) or their shared contribution (PT + MT). However, considering Tcrit across all species, MT explained a larger fraction than PT (with adjusted R^2^ of 0.17 and 0.11, respectively), with PT + MT accounting for the largest amount of variation (adjusted R^2^: 0.64, reflected in a shared fraction of 0.36). Individual partition fractions followed the same trends. Conversely, T50 observed across all species was found to be better explained by PT rather than MT (with adjusted R^2^ of 0.14 and 0.03, respectively), again showing the shared contribution of PT + MT as the partition with the highest explicative power (adjusted R^2^: 0.19), albeit reflected by a low shared partition fraction (resulting R^2^: 0.2).

**Fig. 4:**
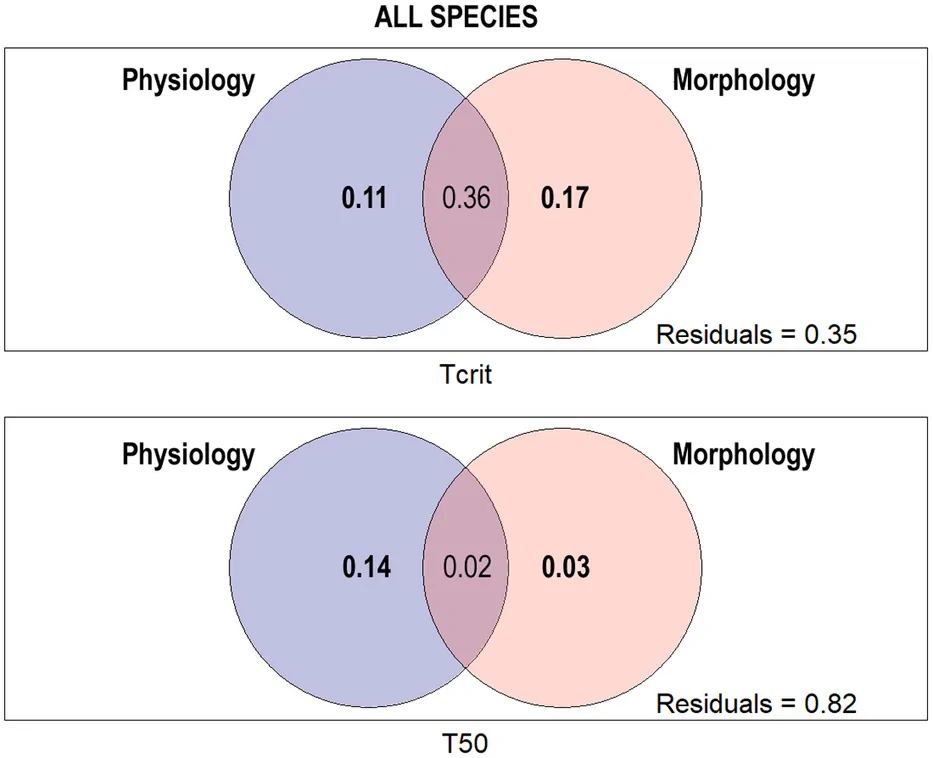
Venn diagram illustrating the partitioning of explained variance in thermal tolerance indices (Tcrit and T50) into unique and shared fractions attributable to morphological and physiological traits across the three study species (*Acer campestre*, *Fraxinus ornus* and *Quercus ilex*).

Overall, these results indicate a predominant role of morphological traits in shaping the early response to heat stress (when approaching Tcrit), with physiological regulation gradually becoming more important in determining thermal tolerance thresholds (approaching T50), keeping in mind that a combination of MT and PT is always a better predictor of variance.

Species‐specific analyses revealed contrasting patterns. In *A. campestre* (Fig. 5), variance in both Tcrit and T50 was primarily explained by PT and by the unique PT fraction (PT|MT), highlighting the dominant role of physiological traits in explaining the variance observed for this species (adjusted R^2^ for Tcrit: PT|MT = 0.44, MT|PT = 0.04; for T50: PT|MT = 0.34, MT|PT = 0.05), with the largest fraction of T50 explained by a high value of shared contribution, which was, however, low for Tcrit (adjusted R^2^ for PT + MT: Tcrit = 0.41, T50 = 0.61; resulting R^2^ of shared partition: Tcrit ~0; T50: 0.22) (Fig. 5). In *F. ornus* (Fig. 6), the largest fraction of explained variance was also associated with the shared contribution (PT + MT), suggesting a coordinated trait syndrome underlying thermal tolerance (resulting shared partition R^2^: 0.42). However, in this species, PT only explained the higher partition of variance for Tcrit (adjusted R^2^ for PT: 0.07; for MT: 0.01), switching to higher explicative power by MT for T50 (adjusted R^2^ for PT: 0.12; for MT: 0.24). The combined fractions showed the best explicative power for *Q. ilex* (Fig. 7) as well (adjusted R^2^ for Tcrit: 0.64; for T50: 0.59) but associated with a low shared partition (~ 0 for both Tcrit and T50), highlighting the independence of these components. Considering individual partitions, a greater proportion of variance was explained by MT and by the unique MT fraction (MT|PT). This indicated that structural leaf traits play a more prominent role in shaping thermal tolerance in this species (adjusted R^2^ for Tcrit: MT = 0.42, PT = 0.23; for T50: MT = 0.46, PT = 0.13).

**Fig. 5:**
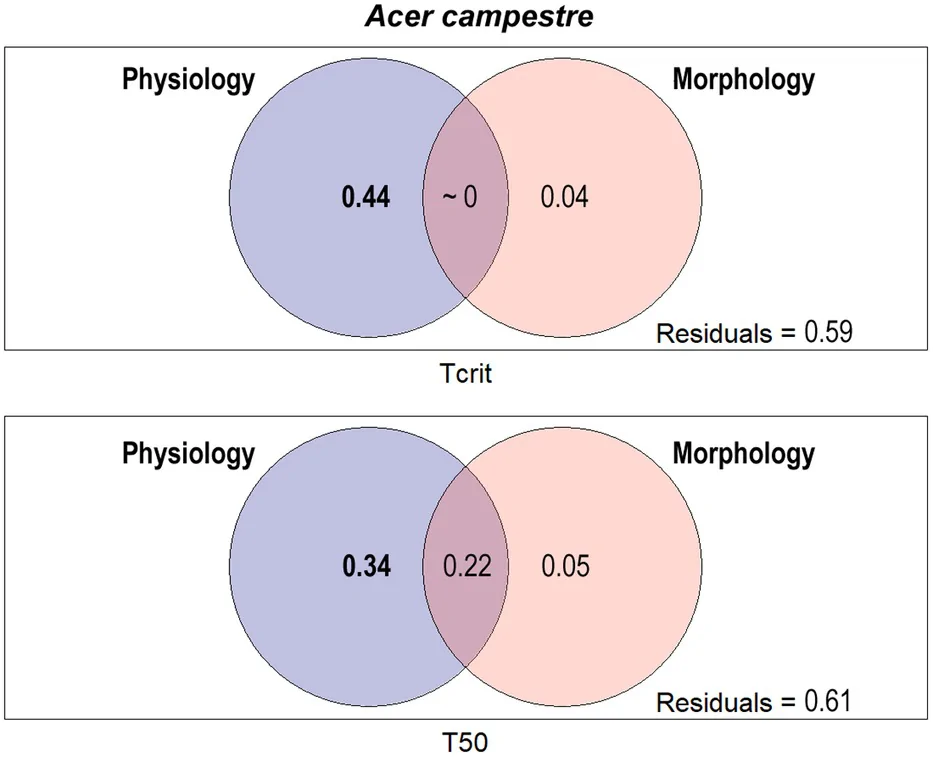
Venn diagram illustrating the partitioning of explained variance in thermal tolerance indices (Tcrit and T50) into unique and shared fractions attributable to morphological and physiological traits across *Acer campestre*.

**Fig. 6:**
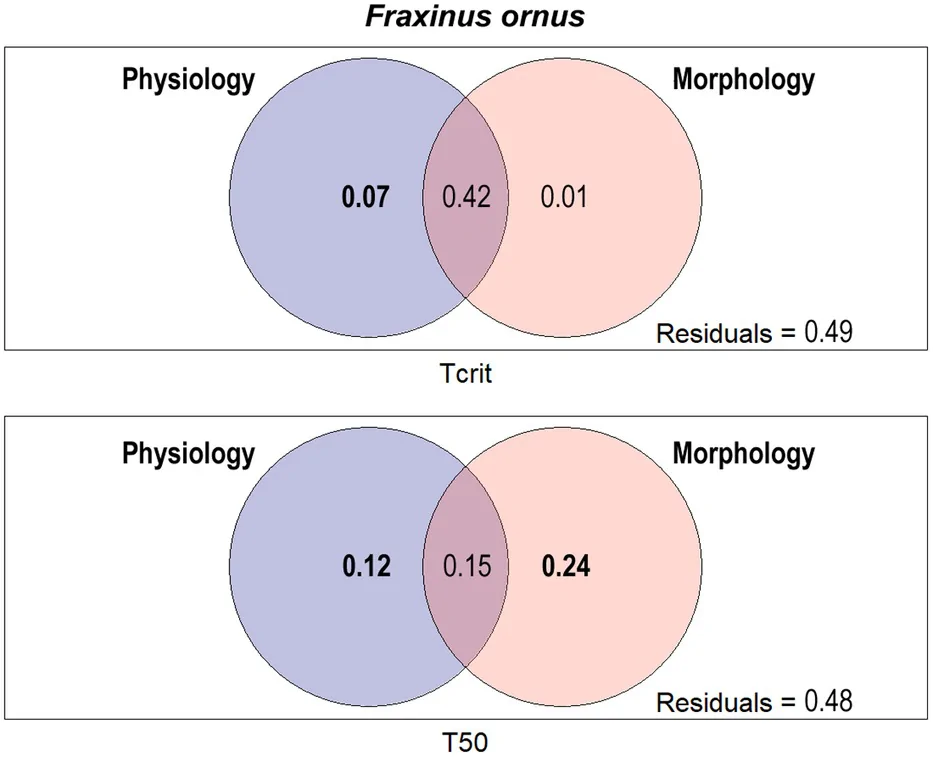
Venn diagram illustrating the partitioning of explained variance in thermal tolerance indices (Tcrit and T50) into unique and shared fractions attributable to morphological and physiological traits across the three study species, *Fraxinus ornus*.

**Fig. 7:**
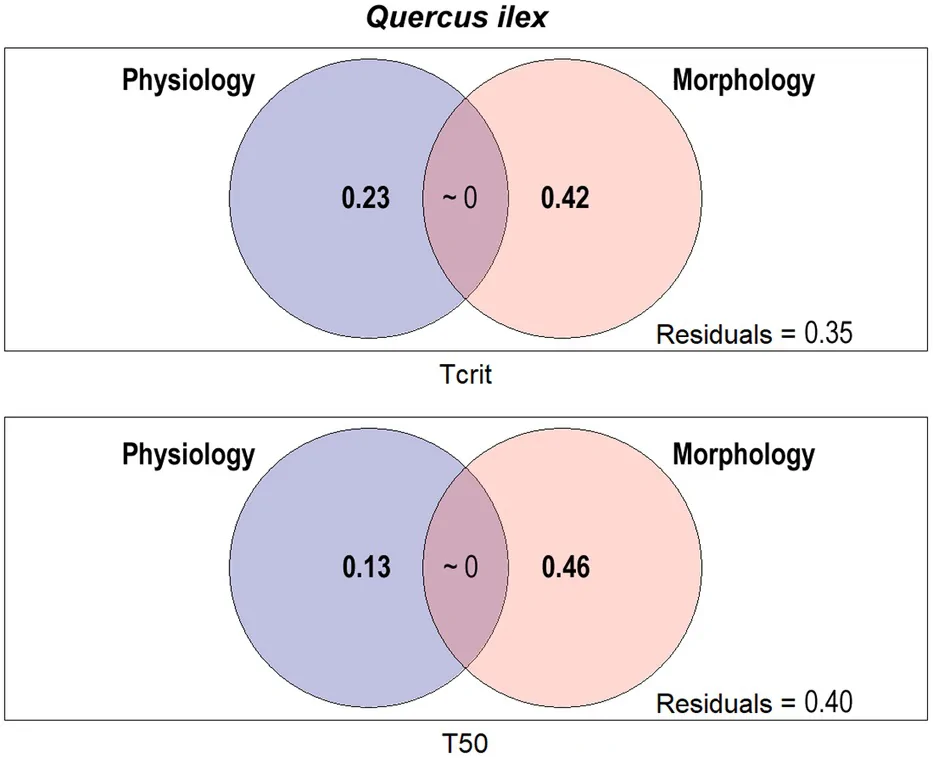
Venn diagram illustrating the partitioning of explained variance in thermal tolerance indices (Tcrit and T50) into unique and shared fractions attributable to morphological and physiological traits across *Quercus ilex*.

## DISCUSSION

Our study provides new insights into how coordinated suites of morphological and physiological leaf traits shape the PSII resistance to thermal stress in Mediterranean tree species with contrasting leaf habits.

Contrary to expectations based solely on structural investment, *Q. ilex* exhibited a significantly lower Tcrit than the deciduous *A. campestre* and *F. ornus*, whereas T50 did not differ among species. At first glance, this pattern appears inconsistent with the greater LT, LTD and LMA that characterize sclerophyllous evergreen leaves and are commonly associated with enhanced stress resistance (Zhang *et al*. 2024). A closer examination of the Fv/Fm temperature response curves reveals that this apparent discrepancy reflects a different species‐specific physiological meaning of Tcrit. In *Q. ilex*, the Fv/Fm decline occurred earlier at relatively lower temperature than in *A. campestre* and *F. ornus*. Nevertheless, with increasing temperature, this decline was less pronounced. This pattern suggests that the estimated Tcrit in *Q. ilex* may not simply represent the onset of irreversible PSII damage. It is consistent with a more conservative strategy characterized by an early activation of PSII downregulation mechanisms that may limit excitation pressure and mitigate irreversible damage. Such a conservative strategy is consistent with the ecophysiology of sclerophyllous species, which, due to a greater leaf thickness and intrinsically lower transpiration rates, frequently operate at leaf temperatures higher than ambient air and thus prioritize damage prevention under recurrent thermal stress (Larcher 2000; Húdoková *et al*. 2022; Münchinger *et al*. 2023). The hypothesis of PSII downregulation is supported indirectly by the significantly lower (more negative) PRI values observed in *Q. ilex*, especially compared with *A. campestre*, indicating a stronger engagement of photoprotective processes. Given that PRI is a well‐established proxy for xanthophyll‐cycle de‐epoxidation and non‐photochemical quenching (Gamon *et al*. 1997; Nakamura *et al*. 2024), and that measurements were carried out in the field under high ambient temperatures (34.1 ± 2.9 °C), close to the estimated Tcrit, it is plausible that Tcrit derived from Fv/Fm response curve may identify the temperature range at which PSII approaches its physiological limits in the field. At this threshold, photoprotective energy dissipation may be favoured over maximal photochemical efficiency to maintain PSII integrity during thermal stress. The persistence of non‐zero Fv/Fm values (i.e. 0.308) at extreme temperatures (above 55 °C) is consistent with the possibility that PSII retains a degree of functional integrity despite the substantial thermal impairment (Rodriguez *et al*. 2012; Bussotti *et al*. 2020). This residual photochemical efficiency suggests that complete thermal inactivation of the photosynthetic apparatus had not yet occurred, even under the most severe temperature treatments.

In addition, leaves with higher structural robustness are known to be less sensitive to extreme heat (Münchinger *et al*. 2023), consistent with the hypothesis that structural robustness may contribute to stabilizing internal structures, including thylakoid membranes, against thermal distortion. This is also in line with our observation of persistent, non‐zero values at the highest temperatures in *Q. ilex*, which showed the highest LMA and LTD. This divergence from the abrupt decline seen in *A. campestre* and *F. ornus* after that Tcrit was reached further supports the interpretation that the threshold in *Q. ilex* reflects a coordinated photoprotective downregulation rather than a direct, irreversible failure of the PSII.

On the contrary, in *A. campestre* and *F. ornus*, Tcrit appears to be a useful indicator of thermal PSII resistance. In our studies, both *A. campestre* and *F. ornus* maintained relatively high PSII efficiency up to Tcrit. However, exceeding this threshold resulted in a rapid and pronounced collapse of Fv/Fm, particularly in *F. ornus*, with values approaching zero at extreme temperatures. This response suggests a limited capacity to sustain PSII functionality once protective limits are surpassed.

While in *Q. ilex*, a lower Tcrit coupled with more negative PRI values is consistent with a photoprotective downregulation strategy; in *A. campestre* and *F. ornus*, the sharp collapse of Fv/Fm after Tcrit suggested the prevalence of thermal damage. Overall, our results are consistent with the hypothesis that structural robustness and early protective downregulation may jointly contribute to PSII thermal stability. Nevertheless, confirming the underlying mechanisms will require dedicated physiological measurements.

PCA further emphasized the results obtained by analysing individual traits, providing an integrated view of how variation in thermal indices was explained by a coordinated leaf trait syndrome.

PC1, which explained a large fraction of the total variance, represented a gradient from acquisitive to conservative leaf strategies, mainly driven by morphological and water‐related traits. Along this axis, *A. campestre* and *F. ornus* clustered in the acquisitive space of the biplot, whereas *Q. ilex* occupied a distinct position in the conservative biplot space.

Tcrit was strongly and negatively correlated with PC1, confirming that species characterized by thicker and denser leaves experienced a lower Tcrit than species with thinner and larger leaves.

T50 showed a significant, yet weak correlation with PC1, but was more strongly correlated with PC3, which was dominated by leaf tissue density. This axis captures variation related to structural resistance to advanced thermal damage, suggesting that denser leaf tissues contribute to maintaining partial PSII functionality once protective mechanisms are exceeded. The persistence of non‐zero Fv/Fm values at extreme temperatures in *Q. ilex*, which exhibited the highest LTD, supports this interpretation, whereas the lower LTD of *F. ornus* is consistent with its more abrupt PSII collapse.

The accumulation of carotenoids and anthocyanins is a good indicator of plant stress, with their abundance reflecting the stress severity (García‐Plazaola *et al*. 1999; Larcher 2000; Yan *et al*. 2022; Dabravolski & Isayenkov 2023). These compounds play a central role in mitigating thermal stress by scavenging reactive oxygen species and dissipating excess excitation energy (Dongsansuk *et al*. 2013; Porcu *et al*. 2025).

*A. campestre* and *F. ornus* primarily relied on physiological adjustments, mediated by carotenoids, as reflected by their markedly higher CRI1 values (about threefold greater than those of *Q. ilex*).

Notably, unlike PRI and ARI, CRI1 was only weakly correlated with PC2, whereas it showed a strong correlation with PC1, indicating a co‐variation with Tcrit. Thus, a high carotenoid content appeared to compensate for limited structural protection in *A. campestre* and *F. ornus*, delaying the onset of PSII impairment at moderate temperatures and contributing to their higher Tcrit values.

Despite this shared strategy, some differences were observed between *A. campestre* and *F. ornus*. In *A. campestre*, the high carotenoid content was accompanied by a greater accumulation of anthocyanins, as highlighted by the ARI1 values approximately four times higher than those of *F. ornus*. In this view, *A. campestre* showed a behaviour similar to *Q. ilex*, as highlighted by the absence of significant differences in ARI1 between these two species.

These findings are consistent with data reported by Dongsansuk *et al*. (2013), who described dynamic shifts in the xanthophyll cycle and β‐carotene under heat stress, with zeaxanthin increasing and violaxanthin decreasing above 30 °C, and β‐carotene increasing above 40 °C (Dongsansuk *et al*. 2013).

The VPA further clarified the relative contributions of physiological and morphological traits in explaining thermal tolerance and supported the patterns emerging from the PCA.

Across all species, morphological traits (i.e. LMA, LT and LTD) accounted for the largest fraction of explained variance in Tcrit. However, these traits represent only a part of the structural strategy adopted by woody species to cope with thermal stress. Cuticular properties, epicuticular waxes, anatomical organization, including mesophyll arrangement, influence leaf energy balance by increasing reflectance, reducing non‐stomatal water loss and limiting internal heat transfer (González *et al*. 2025; Zheng *et al*. 2026). These structural features help maintain leaf thermal safety margins during heatwaves and preserve the integrity of photosynthetic tissues when evaporative cooling becomes ineffective (Fernandes *et al*. 2025; Tiloca *et al*. 2026). Recent studies on woody species further indicate that evergreen and deciduous taxa achieve thermal tolerance through contrasting combinations of anatomical investment and physiological acclimation, reflecting species‐specific responses to environmental conditions (Wang *et al*. 2025). In particular, maintaining cuticle integrity during prolonged heat stress appears crucial to prevent the increase in cuticular water permeability that may compromise long‐term leaf performance (Fernandes *et al*. 2025). Rather than acting independently, these structural traits operate in concert with physiological responses, providing a coordinated structure–function strategy that enhances PSII stability under extreme temperatures. This integrated framework is consistent with the contrasting thermal strategies observed in the woody species analysed here and characterized by contrasting leaf habitus, where different combinations of structural investment and physiological regulation contribute to heat tolerance.

Species‐specific VPAs revealed that, behind this strong coordination of structural and physiological responses, species adopt distinct strategies aligned with differences in leaf habit and resource‐use strategy.

In *Q. ilex*, morphological traits and the unique morphological fraction explained a greater proportion of variance in both thermal indices, suggesting a constitutive structural response to cope with thermal stress.

By contrast, the VPA for *A. campestre* and *F. ornus* shows that these species rely on rapid physiological acclimatization, suggesting that this plasticity may be less effective under extreme heat peaks (Sperlich *et al*. 2019; Porcu *et al*. 2025).

However, the shared fraction (physiological–morphological) resulting from VPA across species confirms that these two levels of response are not independent but represent a coordinated structure–function syndrome. The divergence between the protection‐oriented strategy of *Q. ilex*, that is high structural investment and early PRI‐mediated downregulation is consistent with a strategy that may safeguard PSII stability, and the performance‐oriented strategy of *A. campestre* and *F. ornus*, characterized by lower structural costs and more abrupt damage after Tcrit threshold, highlights the different evolutionary paths taken by Mediterranean trees to cope with extreme heatwaves. While *A. campestre* and *F. ornus* remain more vulnerable to thermal peaks, *Q. ilex* relies on a robust anatomical buffer that contributes to its larger PSII thermal stability. Taken together, these results highlight that the relative contribution of physiological and morphological traits to thermal tolerance is both metric‐dependent and species‐specific.

An additional contribution of this study lies in evaluating the relative sensitivity of thermal tolerance indices. Across species, Tcrit emerged as a more reliable and informative indicator than T50, especially in *A. campestre* and *F. ornus*, since it captures the point at which plants deploy their functional adaptive strategy, before irreversible damage occurs. Notably, the stability of Tcrit across different measurement methods and exposure durations was demonstrated by Didion‐Gency *et al*. (2025) using species with contrasting bioclimatic origins and leaf characteristics, such as Aleppo pine and Mediterranean cypress. The lack of significant variation in Tcrit among these diverse species suggests that the initial damage to the photosynthetic machinery occurs at a consistent temperature threshold regardless of specific leaf traits. This provides a robust biological justification for our comparative approach and confirms that Tcrit is a reliable indicator for assessing thermal tolerance across Mediterranean tree species (Didion‐Gency *et al*. 2025). By contrast, T50 reflected a stage of advanced injury to PSII, where physiological responses became less interpretable. This finding, consistent with previous reports (Doughty *et al*. 2020; Perez & Feeley 2020; Zhang *et al*. 2024), further underscores the value of Tcrit as a functional benchmark for comparative studies of thermal tolerance. However, absolute T50 values should be interpreted with caution as this indicator appears to be more sensitive to the experimental protocol employed Didion‐Gency *et al*. (2025).

In addition, it is important to emphasize that Tcrit and T50 identified in this study represent the thermal resistance of the PSII, rather than the whole‐plant heat tolerance under field conditions. While our approach provides a standardized comparison of the biochemical and structural robustness of the photosynthetic machinery across species, it does not account for the avoidance strategies that plants employ in the field. In the Mediterranean environment, where heatwaves are frequently coupled with drought, the ability to maintain transpiration cooling is often limited by stomatal closure. Under such conditions, the stability of the photosynthetic apparatus becomes the ultimate determinant of survival, defining the physiological safety margin before irreversible damage occurs (Feeley *et al*. 2020).

On the contrary, a potential limitation of our study is the use of a 15‐min heat exposure, which may not fully account for the cumulative ‘heat‐dose’ experienced during prolonged natural heatwaves (Neuner & Buchner 2023). Recent research has shown that the temperature required to induce irreversible damage and visible necrosis decreases as the exposure duration increases (Kunert *et al*. 2026). Specifically, while T50 and Tmax are highly sensitive to the duration of the stress, Tcrit of PSII is more stable across different exposure times, that is 15 *versus* 30 min (Didion‐Gency *et al*. 2025). Therefore, our results should be interpreted as the intrinsic biophysical limit of the photosynthetic apparatus. While 15 min is sufficient to trigger the initial inactivation of PSII, longer durations under field conditions would likely lower the observed thermal thresholds and exacerbate the development of leaf necrosis, particularly in the more vulnerable species.

In conclusion, our study emphasizes that the PSII resistance is not governed by a single‐trait category; it emerges from the coordinated interplay between structure and function. These insights contribute to advancing our mechanistic understanding of plant thermal strategies and provide practical implications for urban forestry. In Mediterranean cities, progressively more and more exposed to extreme heat, *Q. ilex* may offer resilience through structural robustness, whereas *A. campestre* and *F. ornus* contribute physiological flexibility that may be advantageous under fluctuating stress regimes. Nevertheless, to deepen the mechanistic explanation, future research should include direct measurements of NPQ, anatomical traits and a broader representation of evergreen species.

Overall, our results support further details on the different impacts of heat on different tree species, showing how their life strategies can be fundamental to address urban heat and plan resilient cities.

To integrate both strategies in programmes of urban planning could therefore enhance the stability and adaptability of green infrastructures under future climate scenarios.

## AUTHOR CONTRIBUTIONS

LF collected data and wrote the original proof; LMI carried out statistical analysis; MFC provided technical assistance for laboratory activities; LV conceived, designed the study and wrote the manuscript; LF, LMI, MFC and LV revised the manuscript.

## CONFLICT OF INTEREST

The authors declare no conflict of interest.

## Supporting information


**Fig. S1.** Maximum PSII photochemical efficiency (Fv/Fm) measured during a two‐hour control experiment in *Quercus ilex*, *Acer campestre* and *Fraxinus ornus*. Measurements were performed at 15‐min intervals from T0 to T120 while leaves were maintained under the same conditions used before thermal treatments but without heat exposure. For each species, no significant differences among Fv/Fm values measured at the different time intervals were observed (*P* > 0.05, one‐way ANOVA). Data points represent mean ± SD (n = 4 for each time).
**Table S1**. Results of the variance partitioning analysis (VPA) showing the proportion of variance in thermal tolerance indices (Tcrit and T50) explained by physiological traits (PT), morphological traits (MT) and their shared contribution (PT + MT) across the three examined species. Asterisks indicate statistically significant fractions (*P* ≤ 0.05), as assessed by Monte Carlo permutation tests.
**Table S2**. Results of the variance partitioning analysis (VPA) showing the proportion of variance in thermal tolerance indices (Tcrit and T50) explained by physiological traits (PT), morphological traits (MT) and their shared contribution (PT + MT) across *A. campestre*. Asterisks indicate statistically significant fractions (*P* ≤ 0.05), as assessed by Monte Carlo permutation tests.
**Table S3**. Results of the variance partitioning analysis (VPA) showing the proportion of variance in thermal tolerance indices (Tcrit and T50) explained by physiological traits (PT), morphological traits (MT) and their shared contribution (PT + MT) across *F. ornus*. Asterisks indicate statistically significant fractions (*P* ≤ 0.05), as assessed by Monte Carlo permutation tests.
**Table S4**. Results of the variance partitioning analysis (VPA) showing the proportion of variance in thermal tolerance indices (Tcrit and T50) explained by physiological traits (PT), morphological traits (MT) and their shared contribution (PT + MT) across *Q. ilex*. Asterisks indicate statistically significant fractions (*P* ≤ 0.05), as assessed by Monte Carlo permutation tests.

## Data Availability

Data will be made available on request.

## References

[plb70289-bib-0001] Andrew S.C. , Arnold P.A. , Simonsen A.K. , Briceño V.F. (2023) Consistently high heat tolerance acclimation in response to a simulated heatwave across species from the broadly distributed *acacia* genus. Functional Plant Biology, 50, 71–83. 10.1071/FP22173 36210348

[plb70289-bib-0002] Archibold O.W. (1995) Ecology of world vegetation. Chapman & Hall Ltd, London, UK, pp 500.

[plb70289-bib-0003] Attorre F. , Stanisci A. , Bruno F. (1997) The urban woods of Rome (Italy). Plant Biosystems, 131, 113–135.

[plb70289-bib-0004] Berry J. , Bjorkman O. (1980) Photosynthetic response and adaptation to temperature in higher plants. Annual Review of Plant Physiology, 31, 491–543.

[plb70289-bib-0005] Boehnke M. , Bruelheide H. (2013) How do evergreen and deciduous species respond to shade?–tolerance and plasticity of subtropical tree and shrub species of south‐East China. Environmental and Experimental Botany, 87, 179–190.

[plb70289-bib-0006] Bussotti F. , Gerosa G. , Digrado A. , Pollastrini M. (2020) Selection of chlorophyll fluorescence parameters as indicators of photosynthetic efficiency in large scale plant ecological studies. Ecological Indicators, 108, 105686.

[plb70289-bib-0007] Caudullo G. , de Rigo D. (2016) *Fraxinus ornus* in Europe: distribution, habitat, usage and threats. In: San‐Miguel‐Ayanz J. , de Rigo D. , Caudullo G. , Houston Durrant T. , Mauri A. (Eds), The European atlas of Forest tree species. Publication Office of the European Union, Luxembourg, pp 100–101.

[plb70289-bib-0008] Dabravolski S.A. , Isayenkov S.V. (2023) The role of anthocyanins in plant tolerance to drought and salt stresses. Plants (Basel, Switzerland), 12, 2558. 10.3390/plants12132558 PMC10346810 37447119

[plb70289-bib-0009] Didion‐Gency M. , Gauthey A. , Johnson K.M. , Schuler P. , Grossiord C. (2025) Leaf excision and exposure duration alter the estimates of the irreversible photosynthetic thermal thresholds. Plant, Cell & Environment, 48, 5357–5368. 10.1111/pce.15521 40177719

[plb70289-bib-0010] Dongsansuk A. , Lütz C. , Neuner G. (2013) Effects of temperature and irradiance on quantum yield of PSII photochemistry and xanthophyll cycle in a tropical and a temperate species. Photosynthetica, 51, 13–21.

[plb70289-bib-0012] Doughty C.E. , Keany J.M. , Wiebe B.C. , Rey‐Sanchez C. , Carter K.R. , Middleby K.B. , Cheesman A.W. , Goulden M.L. , da Rocha H.R. , Miller S.D. , Malhi Y. , Fauset S. , Gloor E. , Slot M. , Menor I.O. , Feeley K. , Martinez‐Villa J. , Perez T. , Silva Duque A. , Triviño Gonzalez D. , Duque A. (2020) The thermal tolerances, distributions, and performances of tropical montane tree species. Frontiers in Forests and Global Change, 3, 25.

[plb70289-bib-0053] Feeley K. , Martinez‐Villa J. , Perez T. , Silva Duque A. , Triviño Gonzalez D. , Duque A. (2020) The thermal tolerances, distributions, and performances of tropical montane tree species. Frontiers in Forests and Global Change, 3, 25.

[plb70289-bib-0013] Fernandes V. , Farnese F.d.S. , Arantes B.R. , Silva M.L.F. , da Silva F.G. , Ruiz J.M.T. , Slot M. , Cochard H. , Menezes‐Silva P.E. (2025) Leaf minimum conductance dynamics during and after heat stress: implications for plant survival under hotter droughts. Plant Physiology, 197, kiaf026. 10.1093/plphys/kiaf026 39928584

[plb70289-bib-0014] Fusaro L. , Salvatori E. , Winkler A. , Frezzini M.A. , De Santis E. , Sagnotti L. , Canepari S. , Manes F. (2021) Urban trees for biomonitoring atmospheric particulate matter: an integrated approach combining plant functional traits, magnetic and chemical properties. Ecological Indicators, 126, 107707.

[plb70289-bib-0015] Gamon J. , Serrano L. , Surfus J.S. (1997) The photochemical reflectance index: an optical indicator of photosynthetic radiation use efficiency across species, functional types, and nutrient levels. Oecologia, 112, 492–501. 10.1007/s004420050337 28307626

[plb70289-bib-0016] García‐Plazaola J.I. , Artetxe U. , Becerril J.M. (1999) Diurnal changes in antioxidant and carotenoid composition in the Mediterranean sclerophyll tree *Quercus ilex* (L) during winter. Plant Science, 143, 125–133.

[plb70289-bib-0017] Geange S.R. , Arnold P.A. , Catling A. , Coast O. , Cook A.M. , Gowland K.M. , Leigh A. , Notarnicola R.F. , Posch B.C. , Venn S. , Zhu L. , Nicotra A.B. (2020) The thermal tolerance of photosynthetic tissues: a global systematic review and agenda for future research. New Phytologist, 229, 2497–2513. 10.1111/nph.17052 33124040

[plb70289-bib-0018] González M. , Cuervo‐Gómez M. , Garnica‐Díaz C. , Álvarez‐Flórez F. , Cubillos‐Ariza L. , Giral V. , Salgado‐Negret B. (2025) Thermal tolerance is linked to anatomical but not morphological leaf traits in woody species of Andean tropical montane forests. Functional Ecology, 39, 1537–1549.

[plb70289-bib-0019] Gratani L. , Varone L. (2004) Adaptive photosynthetic strategies of the Mediterranean maquis species according to their origin. Photosynthetica, 42, 551–558.

[plb70289-bib-0020] Gratani L. , Varone L. , Bonito A. (2016) Carbon sequestration of four urban parks in Rome. Urban Forestry & Urban Greening, 19, 184–193.

[plb70289-bib-0021] Húdoková H. , Petrik P. , Petek‐Petrik A. , Konopková A. , Lestianska A. , Strelcová K. , Kmet J. , Kurjak D. (2022) Heat–stress response of photosystem II in five ecologically important tree species of European temperate forests. Biologia, 77, 671–680.

[plb70289-bib-0022] ISTAT (2024) Temperature and precipitation in provincial capitals for the year 2022, time series 2006–2022. https://www.istat.it/tavole‐di‐dati/temperatura‐e‐precipitazione‐nei‐comuni‐capoluogo‐di‐provincia‐anno‐2022‐serie‐storica‐2006‐2022/

[plb70289-bib-0023] Kalapos T. , Csontos P. (2003) Variation in leaf structure and function of the Mediterranean tree *Fraxinus ornus* L. growing in ecologically contrasting habitats at the margin of its range. Plant Biosystems, 137, 73–82.

[plb70289-bib-0024] Katsoulas N. , Elvanidi A. , Ferentinos K.P. , Kacira M. , Bartzanas T. , Kittas C. (2016) Crop reflectance monitoring as a tool for water stress detection in greenhouses: a review. Biosystems Engineering, 151, 374–398.

[plb70289-bib-0025] Knight C.A. , Ackerly D.D. (2002) An ecological and evolutionary analysis of photosynthetic thermotolerance using the temperature‐dependent increase in fluorescence. Oecologia, 130, 505–514. 10.1007/s00442-001-0841-0 28547251

[plb70289-bib-0026] Kullberg A.T. , Feeley K.J. (2024) Seasonal acclimation of photosynthetic thermal tolerances in six woody tropical species along a thermal gradient. Functional Ecology, 38, 2493–2505.

[plb70289-bib-0027] Kunert N. , Düsterhöft E. , Stumpf B. (2026) Heat treatment duration affects in vitro‐induced photosynthetic impairment and development of necrotic leaf tissue in three Mediterranean oak species. Plant Biology, 28, 1256–1267. 10.1111/plb.70181 PMC13175945 41548182

[plb70289-bib-0028] Larcher W. (2000) Temperature stress and survival ability of Mediterranean sclerophyllous plants. Plant Biosystems, 134, 279–295.

[plb70289-bib-0029] Martínez‐Villa J.A. , Paquette A. , Feeley K.J. , Morales‐Morales P.A. , Messier C. , Durán S.M. (2024) Changes in morphological and physiological traits of urban trees in response to elevated temperatures within an urban heat island. Tree Physiology, 44, tpae145. 10.1093/treephys/tpae145 PMC11647596 39541501

[plb70289-bib-0030] Mitchell D. , Schönbeck L. , Shah S. , Santiago L.S. (2025) Leaf drought and heat tolerance are integrated across three temperate biome types. Scientific Reports, 15, 12201. 10.1038/s41598-025-95623-5 PMC11982534 40204802

[plb70289-bib-0031] Münchinger I. , Hajek P. , Akdogan B. , Caicoya A.T. , Kunert N. (2023) Leaf thermal tolerance and sensitivity of temperate tree species are correlated with leaf physiological and functional drought resistance traits. Journal of Forest Research, 34, 63–76.

[plb70289-bib-0032] Nakamura Y. , Tsujimoto K. , Ogawa T. , Noda H.M. , Hikosaka K. (2024) Correction of photochemical reflectance index (PRI) by optical indices to predict non‐photochemical quenching (NPQ) across various species. Remote Sensing of Environment, 305, 114062.

[plb70289-bib-0033] Neri P. , Gu L. , Song Y. (2024) The effect of temperature on photosystem II efficiency across plant functional types and climate. Biogeosciences, 21, 2731–2758.

[plb70289-bib-0034] Neuner G. , Buchner O. (2023) The dose makes the poison: the longer the heat lasts, the lower the temperature for functional impairment and damage. Environmental and Experimental Botany, 212, 105395.

[plb70289-bib-0035] Oke T.R. (1982) The energetic basis of the urban heat island. Quarterly Journal of the Royal Meteorological Society, 108, 1–24.

[plb70289-bib-0036] Peguero‐Pina J. , Sisó S. , Sancho‐Knapik D. , Diaz‐Espejo A. , Flexas J. , Galmés J. , Gil‐Pelegrín E. (2016) Leaf morphological and physiological adaptations of a deciduous oak (*Quercus faginea* lam.) to the Mediterranean climate: a comparison with a closely related temperate species (*Quercus robur* L.). Tree Physiology, 36, 287–299. 10.1093/treephys/tpv107 PMC4885939 26496958

[plb70289-bib-0037] Percival G.C. (2023) Heat tolerance of urban trees − a review. Urban Forestry & Urban Greening, 86, 128021.

[plb70289-bib-0038] Perez T.M. , Feeley K.J. (2020) Photosynthetic heat tolerances and extreme leaf temperatures. Functional Ecology, 34, 2236–2245.

[plb70289-bib-0039] Petruzellis F. , Savi T. , Bertuzzi S. , Montagner A. , Tretiach M. , Nardini A. (2018) Relationships between water status and photosystem functionality in a chlorolichen and its isolated photobiont. Planta, 247, 705–714. 10.1007/s00425-017-2814-5 29170912

[plb70289-bib-0040] Porcu F. , Fusaro L. , Scartazza A. , Traversari S. , Pallozzi E. , Stefanoni W. , Di Palma A. , Iozia L.M.I. , Crescente M.F. , Varone L. (2025) Assessing Mediterranean tree species suitability for urban environments: insights from experimental data including 23 leaf functional traits. Plant Physiology and Biochemistry, 229, 110453. 10.1016/j.plaphy.2025.110453 40902355

[plb70289-bib-0041] Richardson A.E. , Wright I.J. , Lenz T.I. , Zanne A.E. (2014) Sapwood capacitance is greater in evergreen sclerophyll species growing in high compared to low–Rainfall environments. Functional Ecology, 28, 734–744.

[plb70289-bib-0042] Rodriguez E. , Azevedo R. , Costa A. , Serodio J. , Santos C. (2012) Chloroplast functionality assessment by flow cytometry: case study with pea plants under Paraquat stress. Photosynthetica, 50, 197–205.

[plb70289-bib-0043] Schumann K. , Leuschner C. , Schuldt B. (2019) Xylem hydraulic safety and efficiency in relation to leaf and wood traits in three temperate *acer* species differing in habitat preferences. Trees, 33, 1475–1490.

[plb70289-bib-0044] Sperlich D. , Chang C.T. , Peñuelas J. , Sabaté S. (2019) Responses of photosynthesis and component processes to drought and temperature stress: are Mediterranean trees fit for climate change? Tree Physiology, 39, 1783–1805. 10.1093/treephys/tpz089 31553458

[plb70289-bib-0045] Tiloca G. , Buchner O. , Gierlinger N. , Neuner G. (2026) Cuticle water permeability, thickness and microchemistry of conifer seedlings and mature trees at the treeline. Environmental and Experimental Botany, 242, 106313.

[plb70289-bib-0046] Valliere J.M. , Nelson K.C. , Martinez M.C. (2023) Functional traits and drought strategy predict leaf thermal tolerance. Conservation Physiology, 11, coad085. 10.1093/conphys/coad085 PMC10645286 38026794

[plb70289-bib-0047] Wahlsteen E. , Avramidou E.V. , Bozic G. , Mediouni R.M. , Schuldt B. , Sobolewska H. (2023) Continental–wide population genetics and post–Pleistocene range expansion in field maple (*Acer campestre* L.), a subdominant temperate broadleaved tree species. Tree Genetics & Genomes, 19, 15.

[plb70289-bib-0048] Wang D. , Chen H. , Yong‐chuan Y. (2025) The causal and scaling relationships among anatomical traits explain tree mortality in subtropical forests under extremely high temperatures. Global Ecology and Conservation, 62, e03797.

[plb70289-bib-0049] Yan W. , Li J. , Lin X. , Wang L. , Yang X. , Xia X. , Zhang Y. , Yang S. , Li H. , Deng X. , Ke Q. (2022) Changes in plant anthocyanin levels in response to abiotic stresses: a meta–analysis. Plant Biotechnology Reports, 16, 497–508.

[plb70289-bib-0050] Zecchin B. , Caudullo G. , de Rigo D. (2016) *Acer campestre* in Europe: distribution, habitat, usage and threats. In: San‐Miguel‐Ayanz J. , de Rigo D. , Caudullo G. , Houston Durrant T. , Mauri A. (Eds), The European atlas of Forest tree species. Publication Office of the European Union, Luxembourg, e012c65.

[plb70289-bib-0051] Zhang H. , Ning Q. , Li Q. , Yang J. , Huang J. , Wang J. , Zhang J. , Zhao P. (2024) Contrasting heat tolerance of evergreen and deciduous urban woody species during heat waves. Functional Ecology, 38, 1649–1660.

[plb70289-bib-0052] Zheng B. , Ewhea A.S. , Zhang H. , Zheng G. , Guo J. , Li D. , Feng D. , Zhang M. , Yang C. , Jin Y. , Tian B. (2026) Integrated stomatal‐wax traits confer sustained tolerance of Pistacia weinmanniifolia to prolonged dry‐heat environment. Plant Physiology and Biochemistry, 232, 111126. 10.1016/j.plaphy.2026.111126 41691927

