# Supplementary material for: Divergent structure–function coordination shapes leaf thermal tolerance in Mediterranean tree species
Source: Plant Biol (Stuttg). 2026 Sep 14;28(7):2421–32. doi: 10.1111/plb.70289 (PMC13750312; doi:10.1111/plb.70289)
Supplement: Supplementary file 1 [file PLB-28-2421-s001.docx]

**SUPPLEMENTARY MATERIAL**

*Divergent structure–function coordination shapes leaf thermal in Mediterranean tree species*

Luisa Friulla, Lorenzo Maria Iozia, Maria Fiore Crescente & Laura Varone

**Figure S1**. Maximum PSII photochemical efficiency (Fv/Fm) measured during a two-hour control experiment in *Quercus ilex*, *Acer campestre*, and *Fraxinus ornus*. Measurements were performed at 15-min intervals from T0 to T120 while leaves were maintained under the same conditions used before thermal treatments but without heat exposure. For each species, no significant differences among Fv/Fm values measured at the different time intervals were observed (P > 0.05, one-way ANOVA). Data points represent mean ± SD (n = 4 for each time).

**Table S1.** Results of the variance partitioning analysis (VPA) showing the proportion of variance in thermal tolerance indices (Tcrit and T50) explained by physiological traits (PT), morphological traits (MT), and their shared contribution (PT + MT) across the three examined species. Asterisks indicate statistically significant fractions (p ≤ 0.05), as assessed by Monte Carlo permutation tests.

**Table S2**. Results of the variance partitioning analysis (VPA) showing the proportion of variance in thermal tolerance indices (Tcrit and T50) explained by physiological traits (PT), morphological traits (MT), and their shared contribution (PT + MT) across *A. campestre*. Asterisks indicate statistically significant fractions (p ≤ 0.05), as assessed by Monte Carlo permutation tests.

**Table S3**. Results of the variance partitioning analysis (VPA) showing the proportion of variance in thermal tolerance indices (Tcrit and T50) explained by physiological traits (PT), morphological traits (MT), and their shared contribution (PT + MT) across *F. ornus*. Asterisks indicate statistically significant fractions (p ≤ 0.05), as assessed by Monte Carlo permutation tests.

**Table S4**. Results of the variance partitioning analysis (VPA) showing the proportion of variance in thermal tolerance indices (Tcrit and T50) explained by physiological traits (PT), morphological traits (MT), and their shared contribution (PT + MT) across *Q. ilex*. Asterisks indicate statistically significant fractions (p ≤ 0.05), as assessed by Monte Carlo permutation tests.
